# Vascular cognitive impairment: Current concepts and Indian perspective

**DOI:** 10.4103/0972-2327.74254

**Published:** 2010-12

**Authors:** Suvarna Alladi, Subhash Kaul, Shailaja Mekala

**Affiliations:** Department of Neurology, Nizam’s Institute of Medical Sciences, Panjagutta, Hyderabad -500082, India

**Keywords:** Dementia, stroke, vascular cognitive impairment

## Abstract

Cognitive impairment due to cerebrovascular disease is termed “Vascular Cognitive Impairment” (VCI) and forms a spectrum that includes Vascular Dementia (VaD) and milder forms of cognitive impairment referred to as Vascular Mild Cognitive Impairment (VaMCI). VCI represents a complex neurological disorder that occurs as a result of interaction between vascular risk factors such as hypertension, diabetes, obesity, dyslipidemia, and brain parenchymal changes such as macro and micro infarcts, haemorrhages, white matter changes, and brain atrophy occurring in an ageing brain. Mixed degenerative and vascular pathologies are increasingly being recognised and an interaction between the AD pathology, vascular risk factors, and strokes is now proposed. The high cardiovascular disease burden in India, increasing stroke incidence, and ageing population have contributed to large numbers of patients with VCI in India. Inadequate resources coupled with low awareness make it a problem that needs urgent attention, it is important identify patients at early stages of cognitive impairment, to treat appropriately and prevent progression to frank dementia.

## Introduction

Cognitive impairment due to cerebrovascular disease is termed “Vascular Cognitive Impairment” (VCI) and forms a spectrum that includes Vascular Dementia (VaD) and milder forms of cognitive impairment referred to as Vascular Mild Cognitive Impairment (VaMCI).[1] While VaD is the second most common cause of dementia, the milder form VaMCI is much more common. Nearly half of individuals with VaMCI convert to dementia after five years.[2] Vascular cognitive disorders are poised to become the silent epidemic of the 21st century and contribute significantly to mortality, disability, and decreased quality of life.[3]

It is now clear that VCI is not a single entity, but represents a complex neurological disorder that occurs as a result of interaction between vascular risk factors such as hypertension, diabetes, obesity, dyslipidemia, and brain parenchymal changes such as macro and micro infarcts, haemorrhages, white matter changes, and brain atrophy occurring in an ageing brain. Factors that determine progression of milder form VaMCI to dementia are not well understood. Since VCI is amenable to prevention and treatment, there is a pressing need to identify factors that protect or predispose to it.[4]

## Vascular Dementia

Vascular dementia (VaD) is the second most common cause of dementia after Alzheimer disease (AD), and in some Asian countries, it is the most common cause.[5–7] Definition of VaD has undergone many modifications in the past and several diagnostic criteria exist, but in its most constructive use, the term refers to “dementia due to cerebrovascular disease”. While it is very heterogeneous in its causation, clinical course, imaging, and pathology–common factors operating are brain injury caused by vascular disease leading to cognitive impairment severe enough to cause functional impairment.

Three clinicoradiologic subtypes are recognizable in clinical practice: Multi infarct dementia is characterised by recurrent stroke, stepwise course, focal neurological symptoms and signs, and multiple cerebral infarcts on brain imaging. Strategic infarct dementia is characterised by an abrupt onset of memory impairment or behavioural change in association with a single, strategically placed infarct. Sites associated with dementia include basal forebrain, medial temporal, thalamic or parieto-occipital infarcts. Subcortical vascular dementia is increasingly recognized as a frequent cause of VaD. The condition is usually the consequence of hypertension and diabetes causing small-vessel disease, which leads to white matter and deep subcortical gray and white matter demyelination and lacunar infarcts.[8]

A uniform clear profile of cognitive syndrome typical of VaD has not been identified. The reasons are probably related to heterogeneity of patient groups in neuropsychological studies. In comparison to AD, there is a general consensus that episodic memory is more impaired in AD, and that executive/attentional processing is more impaired in vascular dementia, especially in patients with subcortical VaD.[9] Patients with VaD also showed greater impairment in both semantic memory and visuospatial/perceptual function than the patients with AD.[10]

## Subcortical Vascular Dementia

Subcortical vascular dementia encompasses the earlier entities, Binswanger’s disease and lacunar state and is characterised by a dysexecutive syndrome with mild memory loss, early gait disorder, Parkinsonian features, behavioral, and urinary symptoms. Onset maybe insidious, course slowly progressive or stepwise, and strokes may occur. It is thought to be more homogenous and a potential target for clinical trials. Criteria have been proposed for subcortical vascular dementia.[11] A hospital-based study characterizing pattern of VaD in India demonstrated that subcortical dementia was the most common form of VaD, followed by cortical-subcortical dementia.

Criticisms abound regarding the usefulness of “VaD” construct and reasons often cited include its wide clinical, radiological and pathological heterogeneity, the frequent occurrence of a milder disorder, frequent association with AD pathology and etiological significance of “incidental” vascular lesions in patients with dementia. However, recognition that VaD exists as a pure entity is confirmed by neuropathological studies and its frequency in consecutive autopsy series has been found to be quite high.[6,12]

## Concept of Vascular Cognitive Impairment

There is increasing evidence that patients with clinically significant cognitive impairment in association with cerebrovascular disease frequently do not fulfil the traditional criteria of dementia.[11] This lead Hachinski *et al*. to propose the term “Vascular Cognitive Impairment” (VCI) to refer to the spectrum of cognitive impairment that is caused by or associated with vascular factors.[1] To corroborate this entity, there is also evidence that VaD is preceded by a state of mild cognitive impairment (MCI), similar to AD.[13] Nearly half of elderly with mild cognitive problems due to vascular disease converted to dementia after five years in the Canadian Study of Health and Aging.[2] Both Amnestic MCI characterized by memory loss and MCI with multiple cognitive deficits (mcd- MCI) appear to be prodromal for VaD.[14,15] Independent of MCI subtype, a study demonstrated that risk of conversion to dementia was associated with presence of potentially treatable vascular risk factors.[14]

VCI encompasses all cases of cognitive impairment of cerebrovascular origin without requirement for dementia and not requiring prominent memory loss.[1] VCI forms a spectrum that includes VaD, mixed AD with a vascular component, and VCI that does not meet dementia criteria (VaMCI). Vascular cognitive impairment without dementia was the most prevalent form of vascular cognitive impairment among those aged 65 to 84 years in the Canadian Study of Health and Aging.[16] Of 270 patients with TIA/ nondisabling stroke, 56% were found to be cognitively intact, 40% had cognitive impairment no dementia (CIND) and 4% had dementia.[17] Poststroke cognitive impairment of varying severity was observed in half of patients with stroke associated with small artery disease 3 months later.[18] As the condition is preventable to a large extent, it is important to identify patients at early stages of cognitive impairment, to treat appropriately, and prevent progression to frank dementia.[1]

## Cerebrovascular Disease- Dementia Interface

Postmortem pathological studies indicate that up to 34% of dementia cases show significant vascular pathology. Mixed degenerative and vascular pathologies are increasingly being recognised and hospital-based series from developed countries report mixed dementia in one-third of patients.[19,20] Further, both small-vessel disease and AD pathology have been found to be linked to loss of neurons in the Ca1 area of the hippocampus.[21] An interaction between the two pathologies was demonstrated in the Nun study, where lacunar strokes were found to magnify the effects of any given load of AD pathology, and vice versa. The odds ratio for a clinically probable AD diagnosis was 4.7 in the presence of AD pathology alone, but it increased to 16.2 in the presence of a combination of AD pathology.[22] The two pathologies are also found to influence each other’s outcome. Incident stroke was a risk factor significantly associated with increased rate of cognitive impairment in AD.[23]

## Vascular Risk Factors and the Brain

There is also strong evidence that cardiovascular risk factors such as hypertension, diabetes, metabolic syndrome, midlife obesity, and hyperlipidemia are independently associated with an increased risk of cognitive decline and dementia.[6,24] The Cardiovascular Health Cognition Study developed a late-life dementia risk index that included older age, worse cognitive test performance, lower body mass index (BMI), *APOE* _4 allele, MRI findings of white matter disease or ventricular enlargement, internal carotid artery thickening on ultrasound, history of bypass surgery, slower physical performance, and lack of alcohol consumption. Dementia risk within 6 years was 4% in those with low scores and 56% in those with high scores.[25] In the elderly, the Rotterdam scan study demonstrated that higher age, small vessel disease, and cardiovascular risk factors are associated with smaller brain volume, especially WM volume.[26]

Recently, studies have also revealed the importance of silent strokes as risk factors for dementia in the elderly. Silent brain infarcts, i.e., infarcts in individuals without clinical manifestation of stroke are detected in 20% of healthy elderly people and up to 50% of patients in selected series.[27,28] They are associated with subtle deficits in physical and cognitive function that commonly go unnoticed. Moreover, the presence of silent infarcts more than doubles the risk of subsequent stroke and dementia.[29]

## Indian Perspective

Vascular cognitive impairment is a problem close to home. Developing countries have a rapidly ageing population and it is projected that 71% of dementia cases will be in the developing world. VaD is the second most common cause of dementia accounting for 39% of cases,[30] and hence, absolute numbers of VaD, is high in India.[31]

Cardiovascular disease burden is high in developing countries including India and has been attributed to the increasing incidence of atherosclerotic diseases, perhaps due to urbanization, epidemiologic transition and higher risk factor levels, the relatively early age at which they manifest, the large sizes of the population, and the high proportion of individuals who are young adults or middle-aged in these countries.[32] Vascular risk factors has been demonstrated to be strongly associated with MCI in an epidemiologic study from Kolkata.[24] Higher prevalence of vascular risk factors in India is likely to increase burden of VaD and VaMCI.

Stroke, the overt manifestation of cerebrovascular disease is one of the most important risk factors for VaD. Stroke burden is increasing rapidly in developing countries (124% and 107% increases in stroke mortality among men and women in developing countries versus 78% and 56% increases, respectively, in the developed countries). Studies have consistently shown that up to 64% of persons who have experienced a stroke have some degree of cognitive impairment[33] with up to a third developing frank dementia.[34] In a hospital-based study from Hyderabad, of 123 consecutive patients from the Stroke registry evaluated a minimum of 3 months after stroke, 91 (74%) were found to have cognitive impairment- 31% with VaD and 43% with VaMCI. A longitudinal follow-up of 50% of the group over a mean period of 13 months demonstrated that all patients with dementia at baseline continued to have dementia at follow-up and none of the cognitively normal patients worsened. Course of VaMCI was variable-seven patients reverted to normal and one patient progressed to dementia.[35] Inadequate resources and low awareness coupled with growing numbers of patients with VaMCI make it a problem that needs urgent attention on a priority basis.

## Diagnosis

The diagnosis of vascular cognitive impairment requires establishing the presence of cognitive impairment *and* its association with cerebrovascular disease. Identifying the presence and impact of cognitive impairment involves the following steps: reporting of subjective symptoms, objective confirmation by neuropsychological and behavioural assessment, determination of severity of cognitive decline, and its functional impact on ADL. Cerebrovascular disease can be established by the clinical history of a stroke and the presence of focal neurological deficits corroborated by brain imaging. The mechanism underlying the stroke can be identified by the use of appropriate investigations, including ECG, 2D ECHO, extracranial and intracranial vascular imaging, and hematological investigations. The association between stroke and cognitive impairment is thought to be substantiated by a temporal relationship between the two and location of infarct in a region appropriate for cognitive impairment.

Clearly a great degree of variability exists in the method of establishing all these features. This difficulty is evident from the various criteria that have been proposed for the diagnosis of VaD. Majority of them have been devised based on consensus rather than clinical data. The Hachinski ischemic score, a simple clinical tool that performs well in the differentiation between AD and multi-infarct dementia, the purpose for which it was originally designed, but mixed dementia remains difficult.[36] The four sets of criteria currently being used in clinical practice and research include ICD-10, DSM-IV, State of California Alzheimer’s Disease Diagnostic and Treatment Centers (ADDTC), and the National Institute for Neurological Disorders and Stroke–Association Internationale pour la Recherche et a’Enseignement en Neurosciences (NINDS-AIREN) criteria.[37,38] Cognitive impairment and presence of significant cerebrovascular disease are common to all criteria, but the method of establishing their presence and the causal relationship between them is variable. Studies evaluating the validity and reliability of these criteria have demonstrated that the existing criteria are not sufficiently sensitive, use different definitions of dementia, and are not easily interchangeable.[39] In an attempt to resolve this controversy, an attempt has been made to simplify the classification system based on clinical and imaging features. VCI, classified on the basis of clinical and imaging features into VCI without dementia, VaD and mixed degenerative/VaD was found to be useful in a large hospital-based cohort of patients.[40]

In an attempt to harmonise methodology to identify and describe individuals with VCI, particularly in the early stages, the National Institute for Neurological Disorders and Stroke (NINDS) and the Canadian Stroke Network (CSN) developed common standards in clinical diagnosis, epidemiology, brain imaging, neuropathology, experimental models, genetics, and clinical trials to recommend minimum, common, clinical, and research standards for the description and study of vascular cognitive impairment.[41] Using the same standards was thought to help identify individuals in the early stages of cognitive impairment, make studies comparable, and integrate knowledge, thereby accelerating the pace of progress of understanding, preventing and treating VCI.

## Outcome

Vascular dementia shortens life expectancy. A 5-year follow-up study of incident dementia cases[42] found that mortality risk was 3.3-times higher for vascular dementia compared with non-demented people. Nearly half of elderly with MCI due to vascular disease converted to dementia after five years in the Canadian Study of Health and Aging.[2] Further, a follow-up study of people with VCI, AD and ‘No cognitive impairment’, showed that most people with VCI showed readily detectable progression by 30 months and depressive symptoms and impaired judgement progressed more commonly in patients with VCI.[43] In a longitudinal study of VaD due to subcortical lacunar infarcts, progressive cognitive decline was determined mainly by the occurrence of new vascular episodes and severity of the cognitive impairment at baseline, providing evidence that ongoing vascular insult is responsible for progression of disease.[44]

## Treatment

Based on the various pathophysiological mechanisms proposed to underlie VCI, a wide range of management strategies have been tried to treat VCI, including control of risk factors, treating stroke mechanism, treatment of cognitive disorder, management of behavioral symptoms, cognitive retraining, and caregiver support. Attempts to reverse or delay progression of cognitive impairment due to cerebrovascular disease by recent studies have given grounds for hope of treatment of VAD.

Decreased incidence of dementia associated with antihypertensive treatment using nitrendipine and perindopril has been demonstrated.[45,46] The Syst-Eur study found that a calcium-channel blocker–based regimen for the treatment of hypertension reduced the incidence of dementia by 50% in older patients with isolated systolic hypertension studies suggest that the use of lipid-lowering agents lowers the risk of dementia and protects against cognitive decline.[47] Recent evidence suggests that prevention of VCI may be possible through physical activity.[48,49]

In a randomised trial of 325 mg/day aspirin (versus no aspirin) conducted on multi-infarct dementia patients, the group of aspirin patients showed significantly higher cognitive scores than the untreated group.[50] The use of acetylcholinesterase inhibitors is based on the demonstration of existence of cholinergic deficits in pure VaD. Donepezil tested in two double-blind, placebo-controlled trials on patients diagnosed with possible or probable VaD showed significant improvement in cognition (ADAS-cog, MMSE) and global functioning scores.[51] Galantamine, Rivastigmine, and Memantine have also demonstrated improvement in cognition and caregiver.[52,53]

## Conclusions

Vascular risk factors and cerebrovascular disease are now recognized to account for a major proportion of cognitive disorders. Emphasis has shifted from diagnosis of VaD based on restrictive criteria to include a broader spectrum of disease VCI that includes mixed dementia and cognitive impairment with no dementia. Control of vascular risk factors and treatment of milder forms of disease need to form the focus of preventive strategies. Variability exists in burden of VaD, its risk factors, subtypes and outcome in different countries and ethnic populations and elucidating reasons for these differences will help in reducing burden of cognitive impairment due to cerebrovascular disease globally.
